# Knowledge and Attitude of Dental Clinicians towards Light-Curing Units: A Cross-Sectional Study

**DOI:** 10.1155/2021/5578274

**Published:** 2021-06-14

**Authors:** Dana Al-Senan, Fatin Ageel, Amani Aldosari, Haifa Maktabi

**Affiliations:** ^1^Department of Clinical Dental Science, Faculty of Dentistry, Princess Nourah Bint Abdulrahman University, Riyadh, Saudi Arabia; ^2^Department of Restorative Dentistry, Prince Sultan Military Medical City, Riyadh, Saudi Arabia

## Abstract

**Objectives:**

Light curing is crucial when applying composite resin restorations. Complete polymerization of the resin depends on delivering adequate light energy to it. Dental clinicians may be unaware of the importance of proper light-curing techniques. This study aimed at evaluating and comparing the level of knowledge of general practitioners (GPs) and specialists (SPs) regarding light-curing units.

**Materials and Methods:**

An electronic survey was conducted online among GPs and SPs of various specialties, working in the governmental sector in Riyadh, Saudi Arabia. Collected data were analyzed for statistical significance.

**Results:**

310 dentists were included in the study. Nearly half of the GPs (45.9%) and more than half of SPs (56.8%) use light-emitting diode (LED) type light-curing units (LCUs). 36.9% of GPs and 29.6% of SPs were unsure about the type of LCUs they use in their dental clinics. 10.8% of GPs and 8.5% of SPs knew the proper term of the power output of LCU. 52.2% of the GPs and 55.7% of SPs were wrong about advancements in technology of LED LCUs. Regarding the use of radiometer, 48.2% of SPs and 35.1% of GPs had responded wrongly, and 37.7% of SPs and 52.3% of GPs were not familiar with the device, showing a statistical significance (*p*=0.040). There was no statistical significance observed in the responses pertaining to their years of experience, expected for two questions.

**Conclusion:**

Both GPs and SPs displayed inadequate knowledge regarding the use of LCUs. Further educational programs are recommended to spread awareness about the handling of LCUs among dental clinicians.

## 1. Introduction

The use of composite resins is increasing dramatically for anterior and posterior restorations due to its better esthetic outcome, improved longevity, and high mechanical properties [1–3]. Even though these restorative materials have gained popularity over the past few years, the failure rate is higher than anticipated. Several factors affect the polymerization of a composite resin restoration: technical factors such as the irradiance and type of Light-Curing Unit (LCU), material-related factors such as the resin shade and thickness, and operator factors such as the light tip position and exposure time [4].

LCU plays an important role in the clinical success of resin-based composite (RBC) restorations. Inadequate polymerization of RBC adversely affects the resin's physical and mechanical properties, decrease the wear resistance, increase microleakage, lead to recurrent caries, and show color instability [5, 6]. However, some dental clinicians lack awareness of the importance of a proper light-curing technique and its impact on the success of the composite restoration [7].

Quartz-Tungsten-Halogen (QTH), Plasma Arc Curing (PAC), Light-Emitting diode (LED), and Argon laser are the four different blue light curing units that are available nowadays in the market. They are used to activate the photoinitiators in the resin materials by producing photons of light [8, 9]. QTH were well known in the past and had a halogen bulb that needed to be cooled frequently, and a fan that helped disperse the unwanted heat away from the bulb. Although they delivered a broad emission spectrum, they required an exposure time of 30 to 40 seconds to cure a 2 mm increment of resin material, which generated a large amount of heat that affected the life span of the device [10, 11].

PAC lights were introduced in an attempt to reduce the light exposure time. Although they delivered high irradiance, they had multiple tips to provide different power outcomes which created a confusion among clinicians. They were also known to be expensive and difficult to be moved around. On the other hand, argon ion lasers generate very intense emission peaks in the blue spectral region, but they became less popular due to several reasons like nonportability, inability to function on batteries, and being expensive [12].

In the early 1990s, LED were introduced. They were light weighted, battery driven, cordless, and required less maintenance [12]. Three generations of LED are available today. The first and second generations emit blue light in a wavelength between 410 and 470 nm but are unable to properly cure resin material with an alternative photoinitiator other than camphorquinone (the most common photoinitiator in resin materials). A third generation LED was developed to overcome this issue, which emits light at a wavelength between 380 and 515 nm and is capable of photocuring the new photoinitiators which were introduced to allow fabricating lighter shades of RBCs [13].

Different shades of RBCs are available providing a wide selection range for better matching of tooth structures. Manufactures' that recommend increasing the exposure time while using darker shades, support the idea that less light penetrates through composites with more opaque shades [14]. Research also found that when the thickness of the increment increases, the overall radiance exposure is reduced [15]. Increasing the curing time for a longer period than manufacturer's recommendations resulted in higher microhardness values and positive effect on DC (degree of conversion) [16, 17].

Additionally, appropriate infection-control methods must be taken to prevent cross-infection.

A barrier must be used to cover LCUs. However, these plastic barriers will reduce the irradiance from the LCU, so the light exposure time must be increased accordingly [18].

The distance between the light-curing unit tip and the surface of the resin material is one of the important factors to be considered since a reduction in radiance power was observed when the distance was increased [19].

Maintenance of LCUs is required to be done because, with lack of monitoring, its quality diminishes over time resulting from heat buildup within the unit, bulb frosting, tip contamination with remnants of composite resin, and sterilization problems. It is recommended to use a radiometer to measure the power output before each clinical session [20, 21].

The knowledge and awareness of dental clinicians regarding the proper use of light-curing units were addressed in several studies internationally [22–24]. A study was conducted among private clinics in Saudi Arabia which showed that dentists expressed poor knowledge, maintenance, and attitude toward LCUs, with most of them reporting that they did not measure the light intensity output [25]. Alshaafi et al. performed a study to evaluate the light intensity output of QTH and LED curing devices located at eight governmental health institutions in Riyadh, Saudi Arabia. The study found that LCU devices fell below the satisfactory value of light intensity and it was recommended that the regular assessment of LCUs was to be made mandatory [26].

The previous literature has highlighted an issue that needs to be considered to minimize any clinical error with regard to LCUs and inadequate polymerization of RBCs. Large number of dentists in Riyadh are working in governmental hospitals and there is a lack of information pertaining to dental clinician's knowledge and awareness about LCUs. The current study aimed at evaluating the knowledge levels of dental practitioners in governmental jobs in Riyadh, Saudi Arabia, regarding the LCUs, and comparing this information between specialists and general dentists.

## 2. Materials and Methods

A cross-sectional survey was conducted over a duration of two months, among general dentists and specialist of various specialties, working in the governmental sector in Riyadh, Saudi Arabia, and who use LCUs often. Only dental clinicians working in governmental sectors in Riyadh were included in this study. The targeted population size was 500, which resulted in a calculated sample size of 310 dental clinicians. Calculation was performed at the 95% confidence level.

The research approval was obtained from Princess Nourah Bint Abdulrahman University Institutional Review Board with the reference number H-01-R-059.

A questionnaire, hosted on the “Google Forms” platform, was distributed electronically via e-mail, social media channels, and personal contacts to dentists. The questionnaire comprised of eighteen questions over three sections. The first section entailed the demographic data including age, gender, type and location of practice, qualification, and years of experience. The second section comprised five questions covering LCU types and their latest advancement, proper positioning technique, and irradiance. It also included two questions covering material science, i.e., the effect of insufficient radiant exposure on RBC properties, and how to overcome any reduction in power output. The third section included four questions related to maintenance, safety, and infection control of the light-curing units, i.e., whether they inspect LCUs before clinical use, the use of radiometer to monitor output, the use of eye protection, and techniques of infection control.

Validation of our questions was performed by inviting 5 experts to validate each question of the survey over a scale of 4 rank: the question is not relevant, the question is somewhat relevant, the question is quite relevant, or the question is highly relevant.

Statistical analyses were carried out using SPSS version 26.0 statistical software (IBM Inc., Chicago, USA). Pearson's correlation coefficient was used to compare the categorical responses of knowledge and practice in relation to the study variables. Chi-square test was done to determine the significant difference between a general dentist and a specialist. A *p* value of <0.05 was considered statically significant.

## 3. Results

### 3.1. Demographics

A total of 382 dentists responded. Respondents who did not use LCUs, or who worked in private clinics, or who were from outside Riyadh province were excluded from the statistical analyses (*n* = 72) leaving a total of 310 responses. Out of 310 respondents, there were 111(35.8%) GPs and the 199 (64.2%) SPs. Most of the GPs were in the younger age group ranging from 20 to 29 years (56.8%), whereas 56.3% of SPs were between 30 and 39 years of age. 69.8% of the SPs respondents were female and 30.2% were male. However, 50.5% of the GPs were female and 49.5% were male. Most respondents were working in governmental hospitals and only 22.7% were working in governmental university clinics. Most of the SPs were board certified (45.7%), and, regarding experience, 51.3% of SPs exceeded 10 years of experience (Table 1). Specialists were from different specialties with the highest response from operative dentists (34.2%), followed by AEGD (advanced general dentists) (14.1%), and then prosthodontists (13.5%) (Table 2).

### 3.2. Knowledge of LCUs

No statistically significant difference was observed between GPs and SPs in their responses to the options of four of the six questions on knowledge of LCUs. Almost half of the GPs (45.9%) and more than half of SPs (56.8%) used LED type LCUs. However, 36.9% of GPs and 29.6% of SPs were unsure about the type of LCUs they used in their dental clinics. Only 10.8% of GPs and 8.5% of SPs correctly answered the question about the proper term of the power output of LCU.

52.2% of the GPs and 55.7% of SPs responded incorrectly to the question regarding the advancement technology of LED LCUs. Approximately half of the respondents (45%) of GP and (46.7%) of SPs chose to increase the curing time to overcome any clinical factor that might affect the reduction in power output of the LCUs. However, statistically significant difference was observed in one option of a multiple response question related to properties of resin-based composite materials and knowledge regarding the position between the LCU tip and the resin material surface. 18% of GPs and 8% of SPs had responded incorrectly regarding the materials properties “option high bond strength” which indicates statistically significant difference (*p*=0.008). For the option “hold the light-curing tip as close as possible to the restoration surface,” 78.4% of GPs and 67.8% of SPs had responded correctly which showed statistically significant difference in their responses *p*=0.047 (Table 3).

### 3.3. Infection Control and Maintenance

Among the respondents, 75.7% of GPs and 71.9% of SPs reported that they inspected and cleaned their LCU before use to ensure it was on the correct setting, with no significant difference between the two groups. Furthermore, most of GPs (60.4%) and SPs (68.8%) used disinfectant with clear barrier technique, with no significant difference between them. However, regarding the use of radiometer device, 48.2% of SPs and 35.1% of GPs had responded incorrectly, and 37.7% of SPs and 52.3% of GPs chose “not familiar with this device” as their response, which showed a statistically significant difference in their responses *p*=0.040. Respondents were asked about the type of eye protection from the blue light hazards. For the option of “I look away from the blue light,” 64% of GPs and 46.7% of SPs had responded positively. However, 44.1% and 40.2% of them chose “hand-held light shield” for eye protection which showed a statistically significant difference in their responses (Table 3).

The respondents' knowledge of the LCUs use and light-activated materials was compared in relation to their experience (<5, 5–10, & >10 years). There was no statistically significant difference observed in the responses between the respondents based on their years of experience expected for two questions. The first was regarding the new advanced technology of LEDs. 48.7%, 56.5%, and 59.1% of respondents with <5, 5–10, and >10 years of experience, respectively, answered incorrectly (*p*=0.048). The second question was with the multiple response question regarding the properties of resin composite materials. 96.4% of respondents with >10 years of experience had responded correctly to the option “low mechanical physical properties,” when compared to 87% and 87.1% of respondents with experience of <5 years and 5–10 years (*p*=0.028), and for the option “more bacterial colonization”, 32.7% of subjects with >10 years of experience had responded correctly, while 49.6% and 42.4% of subjects with <5 years and 5–10 years of experience, respectively, had responded positively (*p*=0.037) (Table 4).

## 4. Discussion

This study aimed to compare the knowledge between general dentists and specialists working in governmental hospitals and academic institutes in Riyadh, Saudi Arabia, regarding LCUs, their maintenance, personal protection, and infection control. A previous study conducted in Riyadh investigating the knowledge and attitude among dentists in private clinics reported that clinicians showed poor knowledge, maintenance, and attitude toward LCUs [25]. To the best knowledge of the current authors, there is no study performed in Riyadh targeting dentists who work in the governmental sectors.

A questionnaire was distributed online to dentists who use LCUs, working in Riyadh in governmental hospitals or universities. Half of the general practitioners' age ranged between 20 and 29 years having less than five years of experience, while half of the specialists' age ranged between 30 and 39 years having more than ten years of experiences. The percentages of male/female of the general practitioners were 49.5/50.5% while the specialists 30.2/69.8%.

The type of LCUs most used according to the respondent is the light-emitting diodes (LED) (45.9% GP and 56.8% specialists). This result is in accordance with a study conducted to evaluate dentists' knowledge regarding LCUs in Edinburgh and northern Turkey [22, 24]. It could be attributed to the fact that LED units are lightweight, portable, and more efficient than other types of LCUs [27]. However, less than half of the dentists (47.7% of general practitioners and 44.2% of specialists) were familiar with technicalities of LED units and were well informed of the new advancement. Understanding about LCU's type and its power output is essential to ensure successful polymerization of light-curing material, surprisingly 36.9% of general practitioners were “unsure” about the type they are using along with nearly one-third of the specialists (29.6%) in the present study. This may indicate that practitioners are not attentive enough to LCU product descriptions and do not realize the importance of applying a proper light-curing technique.

Knowledge of the LCU's irradiance and the energy to properly cure composite resin is essential. However, it has been shown in previous studies that a majority of dentists are not aware of this information [22, 23]. The present result is in accordance with the previous studies, wherein only 10.8% of the general practitioners and 8.5% of the specialists were familiar with the radiometric term “irradiance.”

Operator technique is one of the factors that affect the radiant exposure delivery to a restoration. It is well documented that there was an improvement in the total amount of curing energy delivered after students had received the proper instructions on using LCU [28–30]. Positioning the LCU tip as close as possible and perpendicular to the restoration maximizes the amount of energy to RBC [31]. The percentage of the general practitioners and specialists in the present study who reportedly position the LCU tip at a proper distance is 78.4% and 67.8%, respectively, while only a minority of the dentists (36.9% GP and 45.2% specialists) reported that they hold the tip at a proper angle.

Determining whether or not a composite restoration is completely cured is one of the largest challenges in clinical practice [32]. In this study clinicians were asked how to overcome any clinical factor that might affect the reduction in power output. 45% and 46.7% of general practitioners and specialists, respectively, choose to “increase the curing time more than the manufacturer's recommendation.” Several studies indicate that increasing the light-curing exposure time results in higher overall radiant exposure reaching the RBC layer, thus obtaining better polymerization [16, 33–35]. On the other hand, 44.1% of general practitioners and 48.7% of specialists responded incorrectly by choosing to store RBC material in a refrigerator before clinical application. This is a recommended step when using self-curing composite material [32].

Clinicians must understand the principles of light-curing because improper curing of composites can dramatically affect its physical and chemical properties [36]. This study found that the majority of general dentist (87.4%) and specialists (92%) had appropriate understanding of material-related science regarding insufficient radiant exposure on the mechanical and optical properties of RBC.

The study revealed that the majority of general dentists (75.7%) and specialists (71.9%) inspect and clean the LCU before use and ensure that it is on the correct setting, in good working order, and free of defects and debris, which is in accordance with one of the light-curing guidelines for practitioners [37]. The result is in contrast with Kopperud et al.'s study which showed that only 26.0% of the respondents did visual checks of the lamp's lens or light guide for scratches, spots, or foreign bodies [23].

At the same time, when the clinicians were asked if they used a radiometer to monitor their LCU output before any clinical session, an affirmative response was obtained from only 12.6% and 14.1% of general practitioners and specialists, respectively. A little over one-third of the GPs (35.1%) did not use radiometer devices to check the LCU, while 52.3% were unfamiliar with the device.

Similarly, the responses for the specialists were also alarming, wherein 48.2% were not using the device and 37.7% were unfamiliar with it. These results are in accordance with a study designed to evaluate light intensity output of LCUs used in private clinics in Riyadh, where it was reported that most of the dentists did not measure the light intensity output [25].

Several studies, worldwide, showed that many offices have been delivering inadequate energy when using LCUs [26, 28, 38–43]. Some of the studies were conducted in different areas in Saudi Arabia including governmental institutes, dental schools, and private clinics [25, 26, 44, 45]. All concluded and agreed that regular assessment and standardized maintenance protocol for LCUs are recommended to ensure clinically acceptable outcome from the devices [26, 44, 45]. This suggests that a large portion of dental clinicians in the present study show a high possibility for incorrect curing procedure and signifies that they do not follow the proper guidelines before using LCUs. A possible explanation for those practitioners who do not use a radiometer is that those who work in public clinics were probably not involved in LCU selection and purchase and that light measurement could be performed by others, e.g., dental assistant, radiation safety officer, or the unit manufacturer, without their knowledge. In addition, some LCUs have color indicators to reflect the value of the irradiance without a numerical value, so the unit can be monitored to some extent.

The use of eye protection is mandatory when using LCUs since they emit blue light, which has been proved to have negative effects on the retina [46–49]. It is recommended by all LCU manufacturers to use protective shields or eyewear to block and minimize the bright blue light [46]. Most of the general practitioners' responses (64%) regarding this matter was that “they are looking away from light” during curing and 46.7% of specialist do the same. The result is in agreement with a study performed in Norway among dentists in the public dental service where one-third of the dentists showed insufficient eye protection when light-curing restorations [46]. The explanation for the present result is either most of the dentists look away from the light even after wearing eye protection or using shield or simply they are not aware of the consequences of moving the light away from the restoration. Looking away from the light and from the material to be cured is not recommended since it will reduce the amount of energy delivered if the light position is altered, and it is preferable that the operator should observe the site while wearing eye protection [22].

The most common methods of maintaining the sterility of LCUs are by using an autoclave, wiping with a disinfectant, or using a clear barrier. The current study revealed that the majority use a clear barrier to have the least negative effects in the power value, 60.4% of general practitioners and 68.8% of specialists. The percentage of the clinicians who use autoclaving is 27.9% GPs and 21.6% SPs, and those who use disinfectant was the lowest, 11.7% GPs and 9.5% for SPs. Using of clear barrier was the most common method in the governmental clinics and could be due to its convenience and noninvasiveness and that it is a cost-effective alternative compared to other techniques.

In general, there is no significant relation found between experience (years in practice) and knowledge of LCUs and material science. This finding is supported by earlier studies. Santini and Tuloglu assessed knowledge of material science among a group of dentists and found no positive relationship between years in practice and material science [22, 24]. Ueda compared dentin bonding effectiveness of self-etch bonding cement applied by dental students and experienced clinicians, and no discernible relation was found [31].

Regarding the present study, it is considered to have a low response rate in certain specialties. One explanation is that perhaps some specialists felt less confident or lacked the knowledge regarding the subject and therefore decided against responding. A possible explanation for operative dentists having the highest response rate could be the frequent use of LCUs in their daily practice.

Within the limitation of the present study, it was found that general practitioners and dental specialists possess insufficient knowledge of LCUs and inadequate information about LCU maintenance and eye protection. Generally, there was no significant difference in knowledge among dentists in relation to their years of experience. Based on the present findings, it can be stated that there is an immense need for educational programs, training, and guidance toward LCUs.

## Figures and Tables

**Table 1:** Distribution of demographic data of general dentists and specialists.

Item	General dentist (*n* = 111)	Specialist (*n* = 199)
	No. (%)	No. (%)
*Age groups (in years)*		
20–29	63 (56.8)	14 (7.0)
30–39	40 (36.0)	112 (56.3)
40–49	8 (7.2)	47 (23.6)
>50	0 (0.0)	26 (13.1)
*Gender*		
Male	55 (49.5)	60 (30.2)
Female	56 (50.5)	139 (69.8)
*Type of practice*		
Governmental	84 (75.7)	157 (78.9)
University clinics	27 (24.3)	42 (21.1)
*Qualification*		
DDS/BDS	109 (98.2)	2 (1.0)
Diploma	1 (0.9)	22 (11.1)
MSc	1 (0.9)	51 (25.6)
PhD	0 (0.0)	33 (16.6)
Board certificate	0 (0.0)	91 (45.7)
*Experience (in years)*		
<5	77 (69.4)	38 (19.1)
5–10	26 (23.4)	59 (29.6)
>10	8 (7.2)	102 (51.3)

**Table 2:** Distribution of specialist dentists in different specialties.

Specialties	No.	%
Prosthodontics	27	13.5
Endodontics	22	11.1
Periodontics	3	1.5
Orthodontics	26	13.1
Operative dentistry	68	34.2
Paediatric dentistry	19	9.5
Family dentistry	4	2
AEGD	28	14.1
Oral surgery	2	1
Total	199	100

**Table 3:** Comparison between general dentists and specialists regarding the knowledge towards light-curing unit (LCU) and light-activating materials, maintenance, and infection control.

Item	General dentist (*n* = 111)	Specialist (*n* = 199)	Χ^2^-value	*p* value
	No. (%)	No. (%)		
*1. Knowledge towards light-curing unit (LCU) and light-activating materials*				
1. What type of LCU are you using?				
Quartz-tungsten-halogen lights (QTH)	13 (11.7)	20 (10.1)	3.583	0.465
Plasma-arc lights (PAC)	4 (3.6)	5 (2.5)		
Argon-ion lasers	2 (1.8)	2 (1.0)		
Light-emitting diodes (LED)	51 (45.9)	113 (56.8)		
Unsure	41 (36.9)	59 (29.6)		
2. What is the proper term to describe the amount of power output of the LCU received over a defined area of resin-based dental material?				
Radiant energy	24 (21.6)	44 (22.1)	1.384	0.709
Radiant power	11 (9.9)	14 (7.0)		
Irradiance	12 (10.8)	17 (8.5)		
Radiant exposure	64 (57.7)	124 (62.3)		
3. Insufficient radiant exposure was found to be associated with which of the following properties of resin-based composite (RBC)^*∗*^				
Low mechanical physical properties	97 (87.4)	183 (92.0)	1.724	0.189
More bacterial colonization	45 (40.5)	84 (42.3)	0.084	0.77
High bond strength	20 (18.0)	16 (8.0)	6.936	0.008
Better color stability	11 (9.9)	12 (6.0)	1.577	0.209
4. Location and morphology of the tooth can affect the position between the LCU tip and the resin material surface. The dentist should always aim to^*∗*^				
hold the light-curing tip as close as possible to the restoration surface	87 (78.4)	135 (67.8)	3.924	0.047
hold the light-curing tip up to 10 mm	19 (17.1)	51 (25.6)	2.938	0.086
position the light-curing tip at 45-degree angle	20 (18.0)	42 (21.1)	0.427	0.514
position the light-curing tip at 90-degree angle	41 (36.9)	90 (45.2)	2.006	0.157
5. What is the new advancement technology in the latest LED which makes it different than other types of LCUs?				
It generates monowavelengths	28 (25.2)	58 (29.1)	0.594	0.743
It generates polywavelengths	53 (47.7)	88 (44.2)		
It has an advance filter and ventilation fan	30 (27.0)	53 (26.6)		
6. To overcome any clinical factor that might affect the reduction in power output, you might need to				
store RBC material in a refrigerator before clinical application	49 (44.1)	97 (48.7)	4.523	0.104
increase the curing time more than manufacturer's recommendation	50 (45.0)	93 (46.7)		
choose darker shade of RBC	12 (10.9)	9 (4.5)		

*2. Maintenance and infection control of light-curing unit (LCU)*				
1. Do you inspect and clean the LCU before use to ensure it is on the correct setting, in good working order, and free of defects and debris?				
Yes	84 (75.7)	143 (71.9)	0.529	0.467
No	27 (24.3)	56 (28.1)		
2. Do you use radiometer to monitor your LCU output before any clinical session?				
Yes	14 (12.6)	28 (14.1)	6.445	0.04
No	39 (35.1)	96 (48.2)		
Not familiar with this device	58 (52.3)	75 (37.7)		
3. What do you use to protect your eyes from “blue light hazards?”^*∗*^				
Red laser safety glasses	19 (17.1)	10 (5.0)	12.289	0.005
Light cure shield	45 (40.5)	81 (40.7)	0.001	0.973
Hand-held light shield	30 (27.0)	76 (38.2)	3.96	0.047
Orange protective glasses	49 (44.1)	80 (40.2)	0.445	0.505
My assistant does the curing	35 (31.5)	55 (27.6)	0.525	0.467
I look away from the blue light	71 (64.0)	93 (46.7)	8.531	0.003
4. Infection-control technique was found to affect the light-curing tips and reduce its irradiance value. What is the technique that has the least negative effect?				
Autoclaving	13 (11.7)	19 (9.5)	2.295	0.317
Use of disinfectant solution	31 (27.9)	43 (21.6)		
Disinfectant with a clear barrier	67 (60.4)	137 (68.8)		

^*∗*^Multiple responses.

**Table 4:** Comparison between general dentists and specialists on their knowledge towards their use of light-curing unit (LCU) and light-activated materials based on their experience.

Item	Experience (in years)			*X* ^2^-value	*p* value
	<5 (*n* = 115)	5–10 (*n* = 85)	>10 (*n* = 110)		
*What type of LCU are you using?*					
Quartz-tungsten-halogen lights (QTH)	14 (12.2)	6 (7.1)	13 (11.8)	14.132	0.078
Plasma-arc lights (PAC)	1 (0.9)	2 (2.4)	6 (5.3)		
Argon-ion lasers	0	2 (2.4)	2 (1.8)		
Light-emitting diodes (LED)	64 (55.7)	53 (62.4)	47 (42.7)		
Unsure	36 (31.3)	22 (25.9)	42 (38.2)		

*What is the proper term to describe the amount of power output of the LCU received over a defined area of resin-based dental material?*					
Radiant energy	22 (19.1)	24 (28.2)	22 (20.0)	6.938	0.327
Radiant power	10 (8.7)	6 (7.1)	9 (8.2)		
Irradiance	12 (10.1)	3 (3.5)	14 (12.7)		
Radiant exposure	71 (61.7)	52 (61.2)	65 (59.1)		

*Insufficient radiant exposure was found to be associated with which of the following's properties of resin-based composite (RBC)* ^*∗*^					
Low mechanical physical properties	100 (87.0)	74 (87.1)	106 (96.4)	7.119	0.028
More bacterial colonization	57 (49.6)	36 (42.4)	36 (32.7)	6.587	0.037
High bond strength	17 (14.8)	12 (14.1)	7 (6.4)	4.598	0.1
Better color stability	7 (6.1)	9 (10.6)	7 (6.4)	1.718	0.423

*Location and morphology of the tooth can affect the position between the LCU tip and the resin material surface. The dentist should always aim to* ^*∗*^					
hold the light-curing tip as close as possible to the restoration surface	79 (68.7)	62 (72.9)	81 (73.6)	0.717	0.678
hold the light-curing tip up to 10 mm	22 (19.1)	24 (28.2)	24 (21.8)	2.374	0.305
position the light-curing tip at 45-degree angle	25 (21.7)	16 (18.8)	21 (19.1)	0.346	0.84
position the light-curing tip at 90-degree angle	48 (41.7)	36 (42.4)	47 (42.7)	0.023	0.988

*What is the new advancement technology in the latest LED which makes it different than other types of LCUs?*					
It generates monowavelengths	32 (27.8)	29 (34.1)	25 (22.7)	9.573	0.048
It generates polywavelengths	59 (51.3)	37 (43.5)	45 (40.9)		
It has an advanced filter and ventilation fan	24 (20.9)	19 (22.4)	40 (36.4)		

*To overcome any clinical factor that might affect the reduction in power output, you might need to*					
store RBC material in a refrigerator before clinical application	47 (40.9)	46 (54.1)	53 (48.2)	4.029	0.402
increase the curing time more than manufacturer's recommendation	58 (50.4)	34 (40.0)	51 (46.4)		
choose darker shade of RBC	10 (8.7)	5 (5.9)	6 (5.5)		

^*∗*^Multiple responses.

## Data Availability

The data used to support the findings of this study can be made available upon request to the corresponding author.
